# Unraveling the 2,3-diketo-l-gulonic acid-dependent and -independent impacts of l-ascorbic acid on somatic cell reprogramming

**DOI:** 10.1186/s13578-023-01160-x

**Published:** 2023-11-30

**Authors:** Lining Liang, Meiai He, Yixin Zhang, Chenchen Wang, Zhaohui Qin, Qian Li, Tingting Yang, Fei Meng, Yusheng Zhou, Haofei Ge, Weining Song, Shiyu Chen, Linna Dong, Qiwen Ren, Changpeng Li, Lin Guo, Hao Sun, Wei Zhang, Duanqing Pei, Hui Zheng

**Affiliations:** 1 Guangdong Provincial Key Laboratory of Stem Cell and Regenerative Medicine, GIBH-CUHK Joint Research Laboratory on Stem Cell and Regenerative Medicine, Guangzhou Institutes of Biomedicine and Health, Chinese Academy of Sciences, #190 Kaiyuan Ave. Science City, Guangzhou, 510530 China; 2 Centre for Regenerative Medicine and Health, Hong Kong Institute of Science & Innovation, Chinese Academy of Sciences, Hong Kong SAR, China; 3 University of Chinese Academy of Sciences, Beijing, China; 4 Guangzhou Laboratory, Guangzhou, China; 5 Joint School of Life Sciences, Guangzhou Medical University, Guangzhou, China; 6 Laboratory of Cell Fate Control, School of Life Sciences, Westlake University, Hangzhou, China

**Keywords:** Asc, Metabolite, DKG, TCA cycle, MET

## Abstract

**Background:**

l-ascorbic acid (Asc) plays a pivotal role in regulating various biological processes, including somatic cell reprogramming, through multiple pathways. However, it remains unclear whether Asc regulates reprogramming directly or functions through its metabolites.

**Results:**

Asc exhibited dual capabilities in promoting reprogramming through both 2,3-diketo-l-gulonic acid (DKG), a key metabolite during Asc degradation, dependent and independent routes. On the one hand, Asc facilitated reprogramming by promoting cell proliferation and inducing the conversion from pre-induced pluripotent stem cells (pre-iPSCs) to iPSCs through DKG-independent pathways. Additionally, Asc triggered mesenchymal-epithelial transition (MET) and activated glycolysis via DKG-dependent mechanisms. Notably, DKG alone activated a non-canonical tricarboxylic acid cycle characterized by increased succinate, fumarate, and malate. Consequently, this shift redirected oxidative phosphorylation toward glycolysis and induced MET. Moreover, owing to its antioxidant capabilities, Asc directly inhibited glycolysis, thereby preventing positive feedback between glycolysis and epithelial-mesenchymal transition, ultimately resulting in a higher level of MET.

**Conclusion:**

These findings unveil the intricate functions of Asc in the context of reprogramming. This study sheds light on the DKG-dependent and -independent activities of Asc during reprogramming, offering novel insights that may extend the application of Asc to other biological processes.

**Supplementary Information:**

The online version contains supplementary material available at 10.1186/s13578-023-01160-x.

## Background

l-ascorbic acid (Asc), commonly known as vitamin C, plays a crucial and versatile role as an essential nutrient, participating in numerous physiological processes. As an electron donor, Asc acts as a powerful reducing agent, safeguarding other vital compounds such as lipids, proteins, and DNA from oxidation by donating its electrons [1]. Furthermore, Asc functions as a vital cofactor for various ferrous (Fe^2+^) and α-ketoglutarate (α-KG) dependent dioxygenases, supporting processes such as prolyl hydroxylation and collagen maturation [2], hydroxylation and stabilization of hypoxia-inducible factor 1α (HIF1α) [3], and the demethylation of histones and DNA [4]. Interestingly, in specific circumstances, Asc may exhibit pro-oxidant behavior, particularly when administered in pharmacological concentrations, thereby opening the possibility for high-dose intravenous Asc to be a potentially beneficial adjuvant in the treatment of certain types of cancer [5–7].

Since the first generation of mouse induced pluripotent stem cells (iPSCs) [8], researchers have diligently explored the underlying mechanisms behind reprogramming while striving to enhance its efficiency. In a significant breakthrough, Dr. Duanqing Pei’s laboratory reported in 2010 that the application of vitamin C not only boosts the iPSCs generation efficiency but also enhances the overall quality of the resulting iPSCs [9]. Subsequent investigations further corroborated and reinforced these findings [10].

The functions of Asc during the reprogramming process from mouse embryonic fibroblasts (MEFs) to iPSCs have been the subject of extensive investigation. Remarkably, by modulating the activities of JHDM1A/1B, two H3K36 demethylases, Asc induces demethylation on H3K36, thereby promoting cell proliferation [11]. Moreover, Asc plays a crucial role in facilitating the conversion from pre-iPSCs to iPSCs by reducing H3K9 methylation at the core pluripotency loci [12]. Furthermore, Asc exerts a promoting influence on mesenchymal-epithelial transition (MET), an initial and indispensable step during reprogramming [9, 13], possibly owing to its vital involvement in collagen formation [14, 15]. Nonetheless, the functions of Asc during reprogramming are complex, as it reduces the activity of HIF1α, which otherwise plays a beneficial role in reprogramming [16, 17]. Intriguingly, while both Asc and ten-eleven translocation methylcytosine dioxygenase 1 (TET1) enhance reprogramming efficiency individually, their simultaneous usage results in a decrease below the basal level [18].

Furthermore, despite the long-recognized importance of Asc, the functions of its metabolites have not received substantial attention until now. In mammals, Asc undergoes oxidation, giving rise to L-dehydroascorbic acid (DHAA), through both spontaneous and enzyme-catalyzed reactions [19]. DHAA, in turn, can be reversed back to Asc through reduction or irreversibly hydrolyzed into 2,3-diketo-L-gulonate (DKG). The degradation of DKG can lead to the formation of L-erythrulose (ERY) and oxalic acid (OXA), or in the presence of H_2_O_2_, it can be transformed into L-threonic acid (THR) and OXA. Notably, the presence of H_2_O_2_ can sometimes give rise to a transient formation of a five-carbon intermediate known as 3,4,5-trihydroxy-2-ketopentanoate [20].

To gain deeper insights into the distinct functions of various Asc metabolites, we conducted a comprehensive investigation to determine their respective influences on critical aspects of cell biology. Specifically, we examined their effects on cell proliferation, iPSCs generation from both MEFs and pre-iPSCs, cellular energy metabolism, and MET using a somatic cell reprogramming model.

## Results

### Both DHAA and DKG promote reprogramming

L-ascorbic acid sodium salt (AscNa) and 2-phospho-L-ascorbic acid trisodium salt (AscPNa) are frequently employed as sources of Asc in cell culture. Notably, AscPNa is preferred due to its superior stability in liquid solutions [21], making it a commonly utilized option for treating cells in various contexts, including somatic cell reprogramming [9]. AscNa releases Asc after undergoing simple hydroxylation, while AscPNa requires the assistance of phosphatase in serum for Asc release (Additional File 1: Figure S1A). Notably, varying concentrations of Asc, AscNa, and AscPNa were employed during somatic cell reprogramming, and all three compounds demonstrated comparable efficacy and potency in promoting the reprogramming process (Additional File 1: Figure S1B). Thus, both AscNa and AscPNa were used in the current studies.

**Fig. 1:**
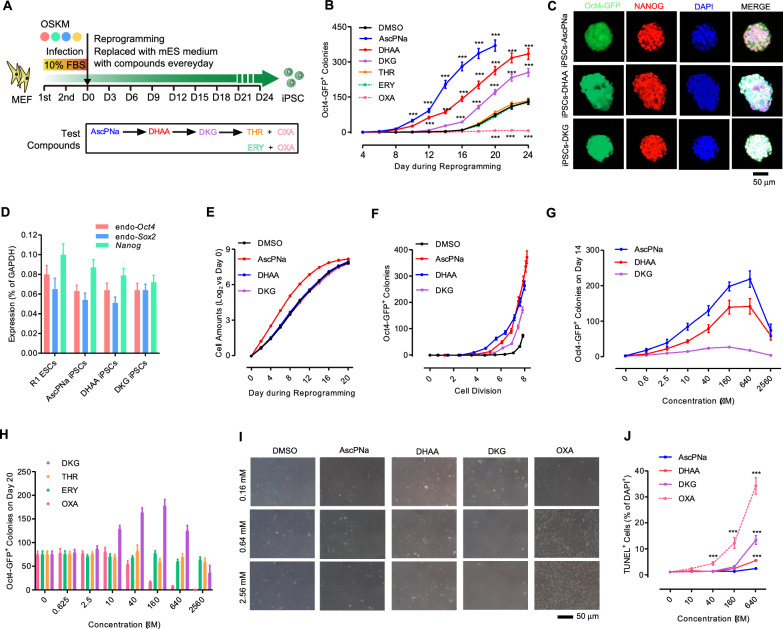
The metabolites of Asc regulate reprogramming differentially. **A** Schematic illustration of the time course of reprogramming with AscPNa, DHAA, DKG, THR, ERY, or OXA. **B** Reprogramming efficiency was assessed by monitoring the number of Oct4-GFP^+^ colonies generated in response to 160 μM AscPNa, DHAA, DKG, THR, ERY, and OXA treatment during reprogramming. **C**–**D** Protein **C** and mRNA **D** levels of pluripotency markers were determined in iPSC colonies generated with DHAA or DKG treatment. **E**–**F** The effects of 160 μM AscPNa and DKG on cell amounts **E** and the correlation between the number of Oct4-GFP^+^ colonies and cell cycle divisions **F** during reprogramming. **G**–**H** Different concentrations of AscPNa, DHAA, DKG, THR, ERY, and OXA were used during reprogramming, and the number of Oct4-GFP^+^ colonies was assessed on Day 14 with AscPNa and DHAA **G** and on Day 20 with DKG, THR, ERY, and OXA **H**. **I**–**J** The impact of different concentrations of AscPNa, DHAA, DKG, and OXA on cell proliferation **I** and apoptosis induction **J** in MEFs. All experiments were conducted at least five times (n ≥ 5). Statistical significance is indicated as follows: * *P* < 0.05, ** *P* < 0.01, and *** *P* < 0.001 compared to the control group or between the indicated groups. Error bars represent standard deviation. Additional statistical information is provided in Additional file 4: Table S3. For related information, see also Additional file 1: Figure S1

The degradation pathway of Asc in mammals involves a series of steps, including oxidation to L-dehydroascorbic acid (DHAA), subsequent hydroxylation to DKG, and eventual degradation to ERY and OXA, or THR and OXA (Additional File 1: Figure S1A) [19, 22]. For the reprogramming study, we utilized AscPNa, DHAA, DKG, ERY, THR, and OXA at a concentration of 160 μM (Fig. 1A). Surprisingly, among these metabolites, only AscPNa, DHAA, and DKG displayed significant promotion of somatic cell reprogramming (Fig. 1B).

Moreover, the Oct4-GFP^+^ colonies generated in the presence of DHAA or DKG exhibited normal karyotypes, typical pluripotent stem cell (PSC) morphologies, and robust expression of key pluripotency markers, such as *Nanog*, endogenous *Oct4*, and endogenous *Sox2* (Fig. 1C–D and Additional File 1: Figure S1C–D).

Both AscPNa and DHAA accelerated the reprogramming process, leading to the attainment of plateau phases for Oct4-GFP^+^ colonies on day 20. In contrast, the control, DKG, ERY, and THR groups reached their plateau phases on day 24 (Fig. 1B). Specifically, on day 14, AscPNa, DHAA, and DKG induced Oct4-GFP^+^ colonies at levels approximately 64, 44, and 9 times of those in the control group, respectively. However, by day 20, these inductions were reduced to approximately 5, 4, and 2 times of those in the control group (Fig. 1B). To ascertain that the observed effects were not influenced by differential cell proliferation in the different groups, we plotted the numbers of Oct4-GFP^+^ colonies against the cell divisions (Fig. 1E–F). Consequently, it was confirmed that DKG promotes reprogramming without affecting cell proliferation.

Next, we investigated the impact of different concentrations of these metabolites on cell reprogramming. AscPNa, DHAA, and DKG exhibited concentration-dependent effects on reprogramming, with the highest efficiencies observed at 160 μM (Fig. 1G–H). However, when used at higher concentrations (2560 μM), these three molecules led to reprogramming suppression (Fig. 1G–H). Conversely, no significant effects on reprogramming were observed when THR or ERY was used (Fig. 1H).

Interestingly, OXA displayed inhibitory effects on reprogramming even at a low concentration (40 μM) (Fig. 1H), possibly due to its ability to impede cell proliferation and induce apoptosis (Fig. 1I–J). Moreover, crystalized oxalate formation was observed at a minimum concentration of 160 μM OXA, with crystal amounts escalating at higher OXA concentrations (Fig. 1I). This observation suggests that crystallized oxalate may underlie the inhibitory effects of high concentrations of AscPNa, DHAA, or DKG on reprogramming, consistent with previous clinical cases where the ingestion of high vitamin C amounts led to oxalate nephropathy [20].

### DHAA promotes reprogramming by providing intracellular Asc and DKG

Given that the oxidation from Asc to DHAA is reversible [19], DHAA treatment could potentially result in increased extracellular and intracellular Asc levels. To investigate this, we initially monitored the concentration of Asc under different conditions. The current kit detected both Asc and ionized Asc. When intracellular Asc concentration or extracellular Asc was mentioned in the following text, the concentration indicated the overall concentration of Asc and ionized Asc. The Asc used to treat cells was pure Asc. Specifically, 160 μM of Asc, AscNa, AscPNa, DHAA, or DKG in mES medium was stored at 4 °C (without cells). For Asc or AscNa, the concentration of Asc gradually decreased from approximately 160 μM to below 70 μM during two days of storage (Additional file 1: Figure S2A). Conversely, for AscPNa, the concentration of Asc peaked at approximately 80 μM after 12 h (Additional file 1: Figure S2A) [23]. Furthermore, DHAA underwent degradation within 12 h and did not yield detectable levels of Asc (Additional file 1: Figure S2A–B).

Subsequently, we examined both extracellular and intracellular Asc levels during reprogramming (day 3). The changes in extracellular Asc mirrored those observed during storage at 4 ℃. Specifically, Asc concentrations gradually decreased in the Asc and AscNa groups, while peaking at hour 8 in the AscPNa group. Conversely, no detectable Asc was generated in the DHAA and DKG groups (Additional file 1: Figure S2C).

**Fig. 2:**
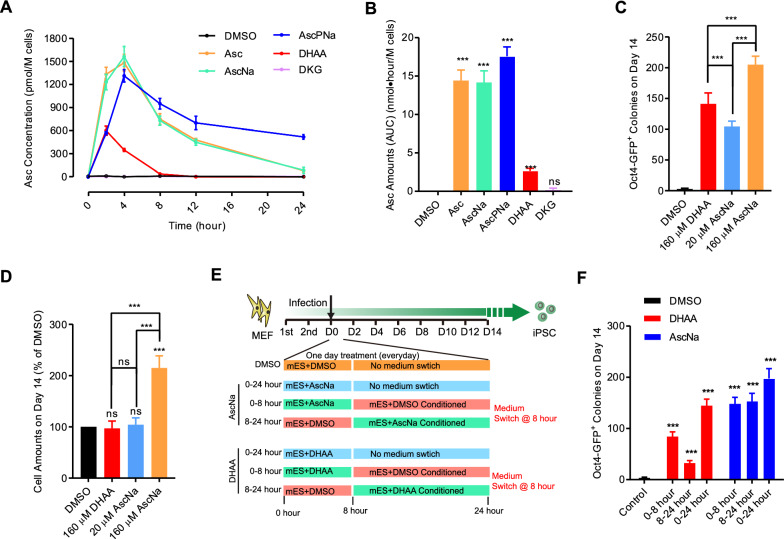
DHAA promotes reprogramming by providing Asc and DKG. **A**–**B** Reprogramming with 160 μM AscPNa, AscNa, Asc, DHAA, or DKG. The intracellular Asc concentration was measured in the 24 h after switching to fresh mES medium at the beginning of day 3 **A**. The accumulated intracellular Asc amounts were calculated **B**. **C**–**D** Reprogramming with DMSO, 160 μM DHAA, 20 μM AscNa, or 160 μM AscNa. The number of Oct4-GFP^+^ colonies **C** and total cell counts **D** were determined on Day 14. **E**–**F** Reprogramming with AscNa or DHAA. The medium was switched with that of the DMSO-treated group on hour 8 daily, dividing the treatment into two phases: 0–8 h and 8–24 h **E**. The number of Oct4-GFP^+^ colonies was determined on Day 14 **F**. All experiments were conducted at least five times (n ≥ 5). Statistical significance is indicated as follows: * *P* < 0.05, ** *P* < 0.01, and *** *P* < 0.001 compared to the control group or between indicated groups. Error bars represent standard deviation. Additional statistical information is provided in Additional file 4: Table S3. For related information, see also Additional file 1: Figure S2

Intriguingly, the dynamics of intracellular Asc showed notable differences. Asc, AscNa, AscPNa, and DHAA significantly increased intracellular Asc levels (Fig. 2A). In the Asc and AscNa groups, intracellular Asc peaked at hour 4. However, in the AscPNa group, the rise in intracellular Asc in the first 4 h was slower, and it declined more gradually in the last 20 h. Interestingly, a small peak of intracellular Asc was identified in the first 4 h in the DHAA group, possibly due to importation from the extracellular medium and subsequent reduction to Asc in the cytosol [19].

Calculation of the accumulated intracellular Asc amounts revealed that 160 μM DHAA provided approximately 16% of the intracellular Asc levels provided by 160 μM Asc, AscNa, or AscPNa (Fig. 2B). To further explore this, we verified that 20 μM AscNa provided a similar amount of intracellular Asc as 160 μM DHAA (Additional file 1: Figure S2D–E). Consequently, we compared their functions during reprogramming. Although both 20 μM AscNa and 160 μM DHAA didn’t influence cell proliferation, 20 μM AscNa promoted reprogramming to a lesser extent than 160 μM DHAA (Fig. 2C–D). Thus, DHAA's promotion of reprogramming is not solely due to its role in providing intracellular Asc.

Additionally, our data in Fig. 1B suggested that DKG can promote reprogramming when used alone. DHAA may facilitate reprogramming by providing DKG. To further validate this, we implemented a switch in the medium for the DMSO and DHAA groups on hour 8 every day (Fig. 2E). Since the intracellular Asc provided by DHAA decreased to basal levels after 8 h, this switch allowed us to dissect the functions of intracellular Asc provided by DHAA (0–8 h group) from the other functions of DHAA (8–24 h group). As depicted in Fig. 2F, reprogramming was still promoted in the 8–24 h group, further confirming that DHAA promotes reprogramming by providing both Asc and DKG.

### DKG promotes reprogramming by inducing MET

As previously summarized in the Introduction, AscPNa has been shown to facilitate cell proliferation, promote the conversion from pre-iPSCs to iPSCs, and induce MET [9, 11–13]. To gain deeper insights into these effects, we conducted a series of experiments aimed at elucidating the specific contributions of DKG to these observed phenomena.

**Fig. 3:**
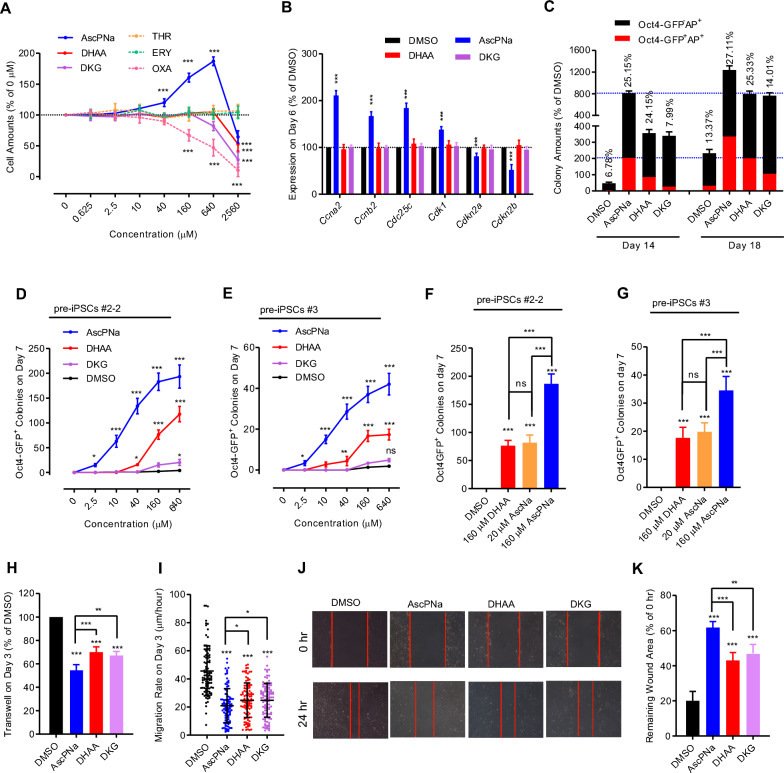
DKG does not affect cell proliferation and pre-iPSCs conversion. **A**–**B** Reprogramming with different concentrations of AscPNa, DHAA, DKG, THR, ERY, or OXA. The number of cells was determined on Day 14 **A**. The expression of key genes related to cell cycle and H3K36 methylation was determined on Day 6 **B**. **C** Reprogramming with 160 μM AscPNa, DHAA, or DKG. The numbers of Oct4-GFP^−^AP^+^ and Oct4-GFP^+^AP^+^ colonies were determined on day 14 and day 18, respectively. **D**-**G** Reprogramming with different concentrations of AscPNa, DHAA, or DKG in Asc-sensitive (#2–2) and Asc-insensitive pre-iPSCs (#3). The number of Oct4-GFP^+^ colonies was determined on Day 7. **H**–**K** Reprogramming with 160 μM AscPNa, DHAA, or DKG. Cell migration on Day 6 during reprogramming was determined using transwell assay **H**, live cell tracing **I**, and wound-healing assay **J**–**K**. All experiments were conducted at least five times (n ≥ 5). Statistical significance is indicated as follows: * *P* < 0.05, ** *P* < 0.01, and *** *P* < 0.001 compared to the control group or between indicated groups. Error bars represent standard deviation. Additional statistical information is provided in Additional file 4: Table S3.

During reprogramming, we employed different concentrations of AscPNa and its metabolites, and the relative cell amounts were assessed on day 14 (Fig. 3A). Among the compounds tested, only AscPNa demonstrated a significant ability to facilitate cell proliferation. Interestingly, high concentrations (2560 μM) of all compounds led to reduced cell amounts, likely attributed to the presence of OXA. Moreover, we evaluated the expression levels of key genes associated with cell cycle regulation and H3K36me3 on day 14 (Fig. 3B). In this analysis, only AscPNa exhibited the capability to induce their upregulation, while DHAA and DKG showed no such effect. Consequently, it became evident that DHAA and DKG promote reprogramming without impacting cell proliferation.

To investigate the potential of these compounds in inducing iPSCs and pre-iPSCs during reprogramming, we determined the number of Oct4-GFP^+^AP^+^ and Oct4-GFP^−^AP^+^ colonies on both day 14 and 18 (Fig. 3C). Although DHAA induced fewer colonies of both types than AscPNa, the percentages of Oct4-GFP^+^AP^+^ colonies were similar. Similarly, DKG induced more colonies than the DMSO control, but the percentages of Oct4-GFP^+^AP^+^ colonies remained similar. Additionally, AscPNa and DHAA exhibited significant abilities to convert two lines of pre-iPSCs to iPSCs (Fig. 3D–E). Notably, these two lines of pre-iPSCs were selected based on their distinct sensitivities to Asc, as reported previously [12].

Remarkably, AscPNa displayed distinct EC50 values for facilitating cell proliferation (approximately 80 μM) and converting pre-iPSCs (approximately 20 μM) (Fig. 1G and 3D–E). This difference in EC50 values partially explains the varying abilities of DHAA in facilitating cell proliferation and converting pre-iPSCs. Furthermore, although 20 μM AscNa did not promote cell proliferation (Fig. 2D), it significantly facilitated the conversion from pre-iPSCs to iPSCs (Fig. 3F–G). Consequently, we can infer that DHAA facilitated pre-iPSCs conversion by providing intracellular Asc but failed to support cell proliferation due to insufficient Asc levels.

Surprisingly, although DKG did not promote pre-iPSCs conversion, it demonstrated a higher ability to induce AP^+^ colonies than the DMSO control (Fig. 3C). Moreover, when we accounted for the influence of cell proliferation by comparing the colony numbers on day 18 in the DKG groups with those on day 14 in the AscPNa and DHAA groups, DKG exhibited a similar ability to induce AP^+^ colonies as AscPNa and DHAA (Figs. 1, 3C). Since cells in AP^+^ colonies exhibited classical epithelial morphology [12], it is reasonable to conclude that DKG had a comparable ability to induce MET as AscPNa and DHAA. Subsequent experiments involving transwell assays, live-cell tracing, and wound-healing further confirmed the abilities of AscPNa, DHAA, and DKG to induce MET (Fig. 3H–K).

### DKG and AscPNa induce MET by regulating similar sets of genes

**Fig. 4:**
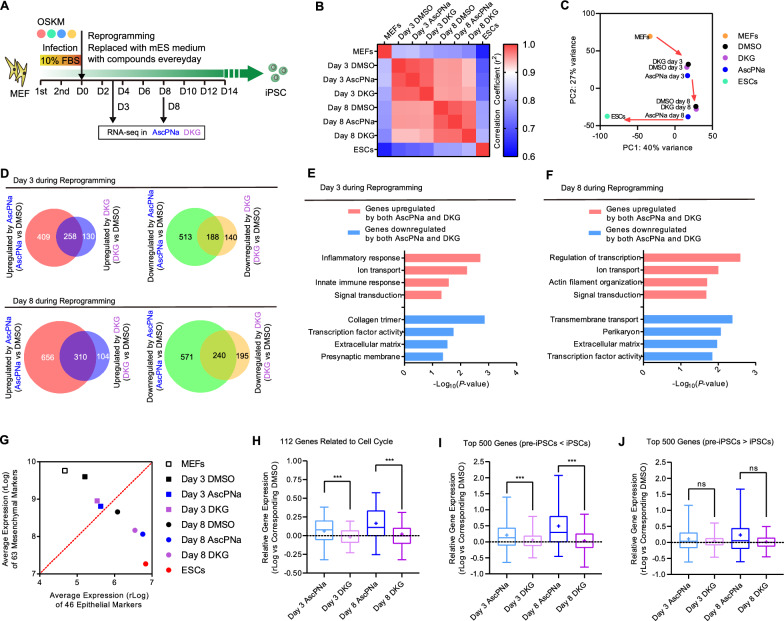
Gene expression profile analysis confirms the functions of DKG. **A**–**C** Reprogramming with 160 μM AscPNa or DKG. RNA-seq analysis was performed on day 3 and day 8 to examine gene expression profiles **A**. The correlation coefficient of gene expression and PCA of the results were provided in **B** and **C**, respectively. **D**–**F** Overlapping genes regulated by AscPNa and DKG. More than 60% of the genes regulated by DKG were also regulated by AscPNa **D**. Gene Ontology (GO) analysis was performed with these overlapping genes **E**–**F**. **G** The expression levels of mesenchymal and epithelial markers in the RNA-seq samples were summarized and plotted to indicate the occurrence of MET. **H**–**J** The abilities of AscPNa and DKG to regulate three groups of genes were summarized. RNA-seq experiments were repeated twice (n = 2). Statistical information is provided in Additional file 4: Table S3.

During the reprogramming process with AscPNa or DKG, we conducted RNA-seq analysis on day 3 and 8 (GSE108695) to gain deeper insights into the molecular changes (Fig. 4A and Additional file 2: Table S1). Notably, we did not include DHAA in this analysis, as it generates both Asc and DKG. Transcriptome analysis effectively validated the reprogramming promoting capabilities of these two compounds (Fig. 4B).

Furthermore, principal component analysis (PCA) revealed that while the reprogramming in the DKG group was relatively slower, the reprogramming route closely resembled that observed in the AscPNa group (Fig. 4C).

We then investigated the regulatory effects of AscPNa and DKG on gene expression. On day 3 during reprogramming, AscPNa upregulated 667 genes, while DKG upregulated 388 genes, with 258 genes showing overlapping upregulation (Fig. 4D). Similarly, AscPNa downregulated 701 genes, and DKG downregulated 328 genes, with 188 genes exhibiting overlapping downregulation (Fig. 4D). These trends were consistent on day 8 as well (Fig. 4D).

Upon conducting Gene Ontology (GO) analysis, we found that the common upregulated genes were enriched with those related to signal transduction and ion transport, whereas the common downregulated genes were enriched with genes related to the extracellular matrix and transcription factor activity (Fig. 4E–F). Subsequently, we analyzed the expression of key markers associated with MET (Fig. 4G), which further confirmed the abilities of both AscPNa and DKG to induce MET during reprogramming.

Next, we focused on 112 genes in the cell cycle pathway (KEGG: mmu10020) (Fig. 4H). AscPNa demonstrated a greater ability to induce expression changes in these genes compared to DKG. Furthermore, we utilized the expression profiles of pre-iPSCs and iPSCs generated in previous datasets (GSE10871 and GSE14012) [24, 25]. Notably, AscPNa exhibited a stronger activation of genes with lower expression in pre-iPSCs compared to iPSCs, but not those with higher expression (Fig. 4I–J).

In summary, the RNA-seq results further supported the previously mentioned conclusion that DKG promotes reprogramming by inducing MET, while its effects on cell proliferation and pre-iPSCs conversion were not significant.

### AscPNa modulates the tricarboxylic acid (TCA) cycle via a DKG-dependent route

As observed in Fig. 3H–K and 4G, the MET induced by DKG was weaker than that induced by AscPNa. Given the positive feedback loop between glycolysis and epithelial-mesenchymal transition (EMT) [26], which opposes MET but may promote reprogramming at the early stage [27, 28], we sought to assess the energy metabolism status of cells treated with AscPNa, DHAA, or DKG.

**Fig. 5:**
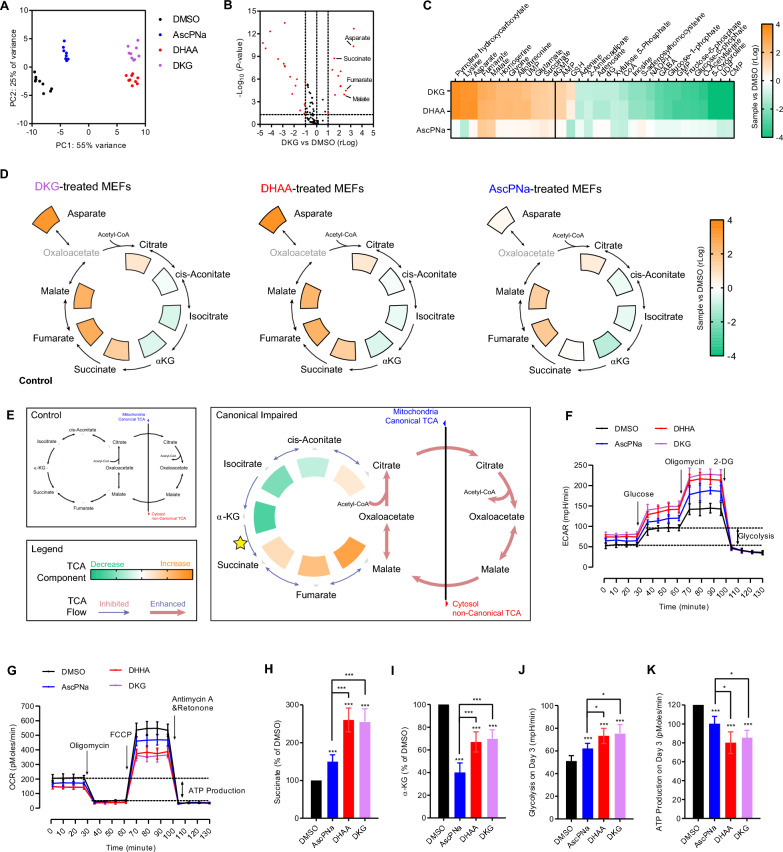
Metabolome analysis suggests the abilities of DKG to regulate TCA cycle. **A** MEFs were treated with 160 μM AscPNa, DHAA, or DKG for three days, and their metabolome profiles were analyzed using 6546 LC/Q-TOF. PCA was performed with the obtained results to visualize the differences in metabolite regulation among the treatments. **B**–**C** Volcano plot showing the compounds regulated by DKG compared to the control **B**. Changes in these compounds by DHAA and AscPNa treatments were summarized **C**. **D** The abilities of AscPNa, DHAA, and DKG to regulate canonical TCA cycle were analyzed. **E** Inhibition of canonical TCA cycle resulted in an increase in succinate, fumarate, and malate, while α-KG levels decreased. **F–G** The effects of 160 μM AscPNa, DHAA, and DKG on ECAR and OCR were determined using the Seahorse instrument. **H**–**K** During reprogramming, MEFs were treated with 160 μM AscPNa, DHAA, or DKG. The concentrations of succinate **H** and α-KG **I**, glycolysis **J**, and ATP production through oxidative phosphorylation (OXPHOS) **K** were determined on day 3. Metabolome analysis was performed at least eight times (n ≥ 8), while other experiments were repeated at least five times (n ≥ 5). Additional statistical information is listed in Additional file 4: Table S3. For related information, see also Additional file 1: Figure S3

Metabolomics analysis was conducted to determine the intracellular concentrations of key components in energy metabolism (Additional file 3: Table S2). PCA indicated that MEFs treated with AscPNa had a metabolome profile similar to that of DMSO-treated MEFs, but distinctly different from that of cells treated with DHAA or DKG (Fig. 5A). Among the 101 metabolites detected in the assays, 32 showed significant differences between the DKG and control (DMSO) groups (Fig. 5B). Comparatively, AscPNa and DHAA induced fewer changes, with 17 and 28 compounds respectively showing significant differences (Additional file 1: Figure S3A-B). Notably, out of the 32 compounds with significant differences in the DKG group, AscPNa modulated 24 of them in the same direction as DKG, albeit to a lesser and statistically insignificant extent (Fig. 5C). This suggests that DKG may be the key component responsible for regulating the metabolome changes observed.

The aforementioned differences in the metabolome were found to be enriched in the TCA cycle or related pathways (Fig. 5B). Notably, the intracellular concentrations of succinate, fumarate, malate, citrate, aspartate, and glutamate increased after treatment with AscPNa, DHAA, or DKG, while the concentrations of cis-aconitate, isocitrate, and α-KG decreased (Fig. 5D). These observed changes in the TCA cycle resembled a previously reported switch from the canonical to non-canonical TCA cycle, where cytosolic citrate is converted to malate and transported to mitochondria [29]. This switch in somatic cells is characterized by increased levels of succinate, fumarate, malate, citrate, and aspartate [29].

The non-canonical TCA cycle bypasses the α-KG, succinate, and fumarate steps in the canonical TCA cycle (Fig. 5E). As these steps are responsible for generating 75% of ATP in the canonical TCA cycle, the switch to the non-canonical TCA cycle impairs ATP production via oxidative phosphorylation (OXPHOS), which may subsequently activate glycolysis. Indeed, we observed decreases in the oxygen consumption rate (OCR) and increases in the extracellular acidification rate (ECAR) in MEFs treated with AscPNa, DHAA, or DKG (Fig. 5F–G). Similar switches to non-canonical TCA cycle and glycolysis were also observed on day 3 during reprogramming (Fig. 5H–K). Consequently, it is reasonable to propose that AscPNa and DKG promote reprogramming by potentially switching energy metabolism from OXPHOS to glycolysis.

### Asc also regulates energy metabolism via a DKG-independent route

While the switch from OXPHOS to glycolysis is advantageous for somatic cell reprogramming [26, 30], our previous report indicated a positive feedback loop between OXPHOS to glycolysis transition and EMT at the early stages of reprogramming [26]. Therefore, the lower ability of AscPNa to activate glycolysis (Fig. 5C–G) might result in less counteraction against MET, which aligns with the high ability of AscPNa to induce MET (Fig. 3H–K and 4G).

It has been proposed that Asc facilitates the canonical TCA cycle in mitochondria by scavenging reactive oxygen species [31], which is supported by the lower levels of α-KG in AscPNa-treated MEFs than in DKG-treated MEFs (Fig. 5D).

**Fig. 6:**
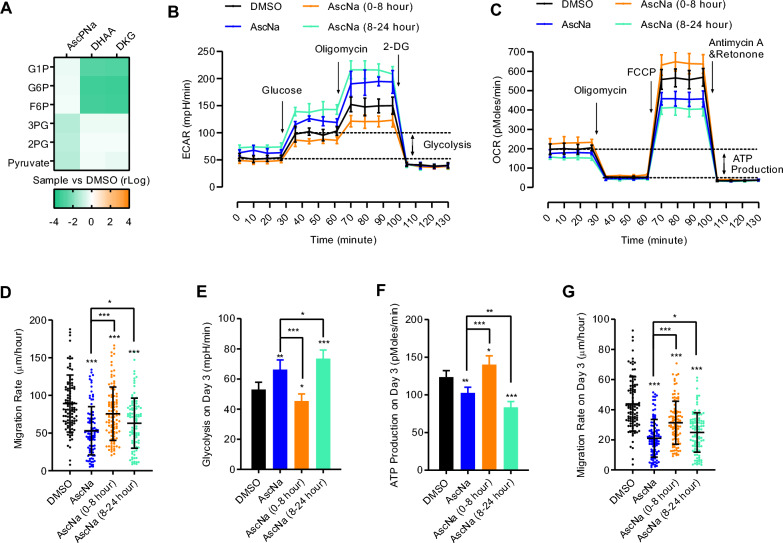
Asc regulates glycolysis via a DKG-independent route. **A** The effects of AscPNa, DHAA, and DKG on key components in glycolysis were analyzed. 2PG: 2-phosphoglycerate; 3PG: 3-phosphoglycerate; G1P: glucose-1-phosphate; G6P: glucose-6-phosphate; F6P: fructose-6-phosphate. **B**–**D** The abilities of the two types of Asc treatment (0–8 h and 8–24 h) to affect ECAR **B**, OCR **C**, and cell migration **D** were determined. **E**–**G** Similar experiments were performed on day 3 during reprogramming as in **B**-**D**. Metabolome analysis was performed at least eight times (n ≥ 8), while other experiments were repeated at least five times (n ≥ 5). Additional statistical information is listed in Additional file 4: Table S3.

Indeed, Asc has been suggested to inhibit the HIF1α pathway [17, 32]. After treatment with AscPNa, but not DKG, several key components in glycolysis, such as 2-phosphoglycerate, 3-phosphoglycerate, and pyruvate, were decreased (Fig. 6A and Additional file 3: Table S2). Moreover, the initial metabolites in glycolysis, including glucose-1-phosphate, glucose-6-phosphate, and fructose-6-phosphate, were decreased by DKG and DHAA, but not AscPNa, treatment (Fig. 6A). As a result, it is reasonable to suggest that AscPNa impairs glycolysis by modulating the route from fructose-6-phosphate to 3-phosphoglycerate, while DKG activates glycolytic flow and reduces the metabolites related to the first and rate-limiting step of glycolysis.

The accumulated amount of Asc in the first 8 h was approximately twice that in the last 16 h in AscNa-treated cells (Fig. 2A). Moreover, the degradation of Asc and DHAA to DKG requires hours [19]. Consequently, the DKG-independent and -dependent functions of Asc were enriched in the first 8 h and the subsequent 16 h, respectively. To further distinguish these two functions of Asc, we divided AscNa treatment into two phases: 0–8 h and 8–24 h as shown in Fig. 2E. When AscNa was only used for the first 8 h, the switch from OXPHOS to glycolysis was reversed (Fig. 6B–C). However, when AscNa was only used for the last 16 h, the switch from OXPHOS to glycolysis was enhanced (Fig. 6B–C). Notably, the cell migration assessed with live-cell tracing suggested that MET was induced to a lower level in the 0–8 h group, while it was enhanced in the 8–24 h group (Fig. 6D).

Cell metabolism and MET were assessed when AscNa was similarly applied during reprogramming, and the observations were consistent with those in MEFs (Fig. 6E–G). Therefore, the DKG-independent pathway was also employed by Asc during reprogramming.

In summary, Asc induces MET and activates glycolysis via a DKG-dependent route while promoting cell proliferation and pre-iPSCs conversion via a DKG-independent route. Furthermore, Asc counteracted DKG-activated glycolysis and subsequently glycolysis-induced EMT (Fig. 6G).

## Discussion

In the current study, we extensively explored the DKG-dependent and -independent functions of Asc during the reprogramming of MEFs to iPSCs. These two distinct routes were also validated in MEFs and are likely applicable to other cell types. The identification and characterization of these routes hold potential benefits for utilizing Asc or related compounds in various biological processes, including cell fate conversion and cancer treatment.

Asc functions as a cofactor for numerous enzymes involved in epigenetic regulation, with many of these functions relying on its antioxidant activity and role as an electron donor. In contrast, DKG lacks similar antioxidant properties, suggesting that it is not involved in these specific functions of Asc. For example, Asc's roles in promoting cell proliferation and pre-iPSCs conversion by regulating H3K9 and H3K36 methylation are independent of DKG.

Asc activates MET and induces the switch from OXPHOS to glycolysis via a DKG-dependent route. However, the underlying mechanism by which DKG induces these downstream effects remains unclear. As Asc is reported to boost the activity of TET, which is important for MET induction during reprogramming [33]. One possibility is that although DKG couldn’t facilitate the reduction of Fe^3+^ back to Fe^2+^ like Asc, it may form a chelator with Fe^2+^ to protect it from oxidation and thereby regulate the intracellular concentration of free Fe^2+^. Our observations that both Asc and DKG delay the precipitation of 1 mM FeCl_2_ in HEPES buffer (pH 7.2) confirmed this hypothesis. We hypothesize that Asc prevents precipitation by inhibiting the oxidation of Fe^2+^ to Fe^3+^, whereas DKG achieves this by forming a chelator with Fe^2+^. If confirmed, this hypothesis suggests that DKG could regulate numerous enzyme reactions related to metabolic and epigenetic regulations through modulation of the intracellular concentration of free Fe^2+^. We truly examined the modulation of DKG on the DNA demethylase TET1, which relies on Fe^2+^ to catalyze the oxidation of 5-methylcytosine. Whole genome bisulfite sequencing showed that DKG reduced DNA methylation level of Tet1-overexpressing MEFs, although to a lesser extent than AscPNa and DHAA (data not shown), confirming the ability of DKG to facilitate TET1’s demethylation activity. Therefore, it is plausible that DKG regulates MET through TET1. It will be of great significance to examine the effects of DKG on TET2 and TET3 activity and resolve the structural basis underlying the selectivity of Asc, DHAA and DKG for these enzymes.

Both Asc and TET1 individually promote reprogramming, while their simultaneous use suppresses reprogramming below basal levels. Since TET1 emerges as a candidate involved in the aforementioned hypothesis, it is plausible to propose that Asc counteracts with TET1 in a DKG-dependent manner, as a similar counteraction has been observed between DKG and TET1 (data not shown). However, further investigation is required to confirm this and explore the underlying mechanisms. Another candidate is aconitase 2 (ACO2), which catalyzes the conversion from citrate to cis-aconitate and isocitrate in a Fe^2+^-dependent manner. DKG's inhibition of canonical TCA cycle, as evidenced by increased succinate, fumarate, and malate, may be attributed to its reduction of the intracellular concentration of free Fe^2+^ and subsequent inhibition of ACO2.

Cell fate determination and conversion are governed by metabolic transition and various epigenetic regulations. As the bridge between glycolysis and OXPHOS, the TCA cycle provides energy and substrates for lipid biosynthesis, and the transition of cell state is accompanied by altered demands for energy and biosynthesis. In rapidly proliferating cells like ESCs and cancer cells, non-canonical TCA is utilized to provide more acetyl-CoA for growth [29]. The promotion of Asc and DKG on non-canonical TCA may induce somatic cells to reach a metabolic state more similar to ESCs. In addition, numerous metabolites of the TCA cycle are involved in these epigenetic modifications. For example, acetyl-CoA and succinyl-CoA provide acetyl and succinyl groups for histone acetylation and succinylation, respectively, while α-KG and FAD^+^ are essential for DNA and histone demethylation [4, 34, 35]. Additionally, succinate and fumarate inhibit the DNA demethylation activity of TETs and Jumonji domain-containing histone demethylases [36].

During the process of reprogramming, primed-to-naive transition, and totipotency acquisition, enzymes of TCA cycle, like PDHA1, PCB, AcCO2, CS, IDH3A, OGDH, SDHA and MDH2, are translocated to the nucleus and regulate the H3 acetylation landscape of chromatin [37]. It is possible that other metabolites, like succinyl-CoA, α-KG and FAD + , also contribute to epigenetic modifications. In addition, the nuclear translocation of substrates and enzymes may also contribute to the reduction of canonical TCA cycle in mitochondria. Although the regulation of Asc and DKG on the nuclear TCA cycle is still unclear, through regulation of the proportion of canonical, non-canonical and nuclear TCA cycle, and the transition between glycolysis and OXPHOS, both Asc and DKG can be implicated in a variety of cellular processes.

## Conclusions

Current studies have unveiled both the DKG-dependent and -independent functions of Asc, illuminating the intricate connections between Asc, epigenetic regulation, and cell fate conversions. These findings offer valuable insights that advance our comprehension of the role of Asc in diverse biological processes.

## Methods

### Materials

The antibodies, chemicals, commercial assay kits, datasets, kits, software, and other reagents and resources are all listed in Additional file 5: Table S4. The authors are willing to distribute all materials, datasets, and protocols described in this manuscript. Further information and requests for resources and reagents should be directed to and will be fulfilled by Hui Zheng (zheng_hui@gibh.ac.cn).

### Cell culture and generation of iPSCs

MEFs used in this study were obtained from E13.5 embryos of OG2/129 transgenic mice [9]. The MEFs and plat-E cells were cultured in high-glucose DMEM (Dulbecco's Modified Eagle Medium) supplemented with 10% fetal bovine serum (FBS), GlutaMax, and non-essential amino acids. iPSCs were generated following the protocol described previously[27]. Briefly, retroviruses encoding *Oct4*, *Klf4*, *Sox2*, and *c-Myc* were generated and collected from the culture medium of plat-E cells following transfection with the corresponding constructs. This retrovirus-containing medium was then used to infect MEFs, delivering *Oct4*, *Klf4*, *Sox2*, and *c-Myc* into the cells. After two rounds of infection, the MEFs were cultured in mES medium, which consisted of DMEM supplemented with 15% FBS, GlutaMax, non-essential amino acids, sodium pyruvate, 2-mercaptoethanol, penicillin–streptomycin, and leukemia inhibitory factor. Derivatives and metabolites of Asc were used to treat cells during reprogramming.

Pre-iPSCs were converted into iPSCs in mES medium with different derivatives and metabolites of Asc. R1 ESCs and iPSCs were cultured on MEF feeder cells in mES medium with PD0325901 and CHIR99021. All cells were subjected to a mycoplasma test (MycoAlert^™^, Lonza) to ensure that they were free of mycoplasma before use.

### Cell proliferation

MEFs were seeded into plates at the concentration of 15 × 10^3^/well. After two infections of reprogramming factors, cells were cultured with mES medium supplemented with Asc, AscNa, AscPNa, DHAA, DKG, THR, ERY and OXA, respectively. For cell proliferation analysis, cells before day 10 of reprogramming were treated with 0.25% trypsin for harvesting, and cells at later stages were pretreated with collagenase IV before dissociation with trypsin. Then, the cells were collected and counted with a hemocytometer.

### qPCR and RNA-seq

Total RNA was extracted from the cells using TRIzol reagent (Thermo Fisher, 15596018), and cDNA synthesis was performed with ReverTra Ace^®^ (Toyobo, TRT-101) and oligo-dT (Takara) according to the manufacturers' protocols, using 2 μg of RNA as starting material. The transcript levels of the target genes were measured using 2 × Master Mix (Vazyme, Q311-02-AA) and a CFX-96 Real-Time system (CFX96, Bio-Rad).RNA was extracted from cells using TRIzol reagent.

For RNA-Seq analysis, RNA was extracted from the cells using TRIzol reagent. RNA-Seq libraries were prepared for each RNA sample using the TruSeq RNA Sample Preparation Kit v2 (RS-122-2001, Illumina). Sequencing was carried out using a NextSeq 500 High Output Kit v2 (75 cycles) (FC-404-1005, Illumina) according to the manufacturer's instructions, and the sequencing depth was set to 10 M pair-end reads of length 50NT. Raw data obtained from RNA-Seq were subjected to basic quality control using the FASTQC tool, and the filtered reads were pre-processed with the TRIMMOMATIC tool (PE.fa:2:30:10:8:true LEADING:3 TRAILING:3 SLIDINGWINDOW:4:15 MINLEN:36). Read alignment to a transcriptome index generated from the Ensembl annotations (v67) was performed using RSEM and bowtie2, with sequencing data using GC-content normalization [38]. Gene Ontology analysis was conducted using DAVID 6.8 (https://david-d.ncifcrf.gov/) [39].

### AP staining and immunofluorescence staining

For AP staining, the cells were fixed with 4% paraformaldehyde for 2 min and then washed twice with PBS. Subsequently, they were incubated with AP staining solution (10 μL/mL nitroblue tetrazolium and 2 μL/mL 5-bromo-4-chloro-3-indolyl in PBS) in the dark at room temperature for 15 min. Afterward, the cells were rinsed with PBS.

For immunofluorescence staining, the cells were fixed with 4% paraformaldehyde after removing the medium and washing three times with PBS. The samples were then washed twice with PBS and blocked with blocking buffer (PBS containing 10% normal goat serum, 1% bovine serum albumin, and 0.3% Triton X-100). Antibodies were diluted with blocking buffer and incubated with the samples overnight at 4 ℃ or 2 h at room temperature. After each antibody incubation, three PBS washes were performed. Immunofluorescence was detected using a Zeiss LSM800 confocal laser scanning microscope.

### Analysis of key components in energy metabolism

MEFs were seeded into 100 mm dishes at 3 × 10^5^/dish, and treated with Vc, DHAA or DKGfor 4 days. After aspirating the culture medium, the cells were washed 2–3 times with PBS and harvested using 0.25% trypsin. Cells were centrifuged at 300 × g for 5 min, resuspended in PBS, counted with a hemocytometer, and centrifuged at 1200 ×*g* for 10 min at 4 ℃. After discarding the supernatant, 50–200 μL of extraction buffer (acetonitrile/methanol/aqueous = 4:4:2, kept at – 20 ℃ overnight before use). The mixture was kept on ice for sonication for 5 min followed by future centrifugation at12000 ×*g* for 5 min. The supernatants were transferred to new tubes and lyophilized. Upon detection, the samples were resuspended, transferred to vials and analyzed with an Agilent 6546 LC/Q-TOF system.

The mass spectrometer was equipped with an Agilent Jet-stream source operating in negative and positive ion mode with source parameters set as follows: nebulizer gas, 45 psi; sheath gas temperature, 325 °C; sheath gas flow, 10 L/min; dry gas temperature, 280 °C; dry gas flow, 8 L/min; capillary voltage, 3500 V for two ion modes and nozzle voltage, 500 V for positive mode and 1000 v for negative mode. The QTOF scan parameters were set as follows: scan speed, 2 scan/s. Metabolites were separated with a Waters ACQUITY UPLC BEH Amide column (2.1 mm × 100 mm × 1.7 μm) and guard column (2.1 mm × 5 mm × 1.7 μm). The elution solvents consisted of A (100% H_2_O) and B (ACN: H_2_O = 90:5, v/v), both containing 15 mM ammonium acetate and 0.3% ammonium hydroxy. The elution gradient was set as follows: 10% A (0.0–8.0 min), 50% A (8.0–10.0 min), 50% A (10.0–11.0 min) and 10% A (11.0–20.0 min). The working column temperature was 35 °C and the flow rate was 0.3 mL/min. The capillary charge was 3.5 kV for the positive and negative modes. Data in both electrospray ionization positive and negative modes were acquired and then analyzed using Profinder 10.0 and Mass Profiler Professional 15.1.

### Seahorse analysis

Cellular energy metabolism was assessed using the Seahorse XF24 extracellular flux analyzer (Seahorse Bioscience) according to the manufacturer’s instructions. with the simultaneous measurement of the OCR and the ECAR as the indicator of mitochondrial respiration and glycolysis, respectively. Cells were seeded into Seahorse XF24 cell culture plates. After cell adhesion, 150 μL culture medium was added to bring the total volume to 250 μL, and the cells were allowed to grow overnight in a cell culture incubator. The sensor cartridge was hydrated in Seahorse XF calibrant at 37 °C in a non-CO_2_ incubator overnight. For mitochondrial respiration, the cell culture medium was replaced with pre-heated XF assay medium supplemented with 25 mM glucose, and the cells were then cultured at 37 °C in a non-CO_2_ incubator for 1 h. For analysis, 1 μM oligomycin and 1 μM FCCP were added at 30 min and 60 min, respectively. Then, 1 μM rotenone and 1 μM antimycin A were simultaneously added at 90 min. For glycolysis analysis, the assay medium was replaced by XF assay medium without glucose or pyruvate. The assay workflow was as follows: 10 mM glucose was injected at 30 min, 1 μM oligomycin at 60 min, and 50 mM 2-DG at 90 min. OCR and ECAR parameters were then calculated. The increase in ECAR after glucose injection indicated the level of glycolysis, while the decrease in OCR after oligomycin injection indicated the level of ATP production or OXPHOS.

### TUNEL assay

Cells were dissociated with trypsin and counted with a hemocytometer. The cells were washed with PBS, and fixed with 4% paraformaldehyde for 15 min. Cells were centrifuged at 300 ×*g* for 5 min to discard the supernatant, then washed with PBS for 3 times. Then, 100 μL of 0.1% Triton X-100 in 0.1% sodium citrate was added to resuspend the cells for 2 min on ice. After permeabilization, the cells were washed twice with PBS, resuspended in 50 μL TUNEL reaction mix and incubated for 60 min at 37 °C in a humidified atmosphere in the dark. The cells were washed twice with PBS, resuspended with 200 μL of PBS, and analyzed by flow cytometry.

### Asc, αKG, and succinate quantification

The concentrations of intracellular and extracellular Asc were measured using an Ascorbic Acid Assay Kit from Abcam (ab65656). To detect the amount of intracellular Asc, the cells were harvested, counted, lysed in cold ddH_2_O, and then centrifuged at 13, 000 g at 4 ℃ for 5 min to remove insoluble materials. For extracellular Asc measurement, medium can be used directly. Standards and samples were added to 96 wells, ddH_2_O was added to bring the volume to 100 μL, 10 μL ddH_2_O was added to each standard and sample well, and 10 μL ascorbate oxidase was added background control wells. A reaction mix consisting of FRASC buffer, ascorbic acid probe and FeCl_3_ was added to all wells. The wells were mixed by pipetting and incubated for 2–3 min. The absorbance at 593 nm was measured in kinetic mode, and the background was corrected by subtracting the value of the well containing ascorbate oxidase. After generating the standard curve, the concentration of Asc was determined.

The contents of intracellular α-KG were measured with an α-ketoglutarate assay kit. Cells were dissociated with trypsin and counted with a hemocytometer. 1 × 10^6^ cells were homogenized with 100 μL ice-cold α-KG assay buffer, and then centrifuged at 13,000 ×*g* at 4 ℃ for 10 min. The supernatant was transferred to a 3 kDa MWCO spin filter to remove the proteins that might interfere with the assay. Standards or samples were added to 1 96-well plate, and αKG- assay buffer was added to each well to bring the volume to 44 μL. For a blank control, 46 μL α-KG assay buffer was added. 2 μL α-KG converting enzyme was added to each standard and sample well, 2 μL αKG development enzyme mix and 2 μL prediluted fluorescent peroxidase substrate were added to each well. Wells were mixed by pipetting, and incubated at 37 ℃ for 30 min away from light. The fluorescence intensity was measured with λex = 535 and λem = 587 nm. All readings were corrected by subtracting the blank value, a standard curve was plotted and the contents of the samples were determined.

The concentrations of intracellular succinate were measured following the instructions of the succinate assay kit. Cells were homogenized in ddH_2_O, then centrifuged at 13,000 ×*g* at 4 ℃ for 10 min to discard precipitates. 20 μL standards and sample supernatant were added to separate wells of 96-well plate, and 80 μL regant mix was added to each well. The plate was incubated at room temperature for 30 min, and the fluorescence intensity was measured with λex = 530 and λem = 585 nm. The contents of the samples were determined with standard curve.

### DHAA quantification with HPLC

DHAA was diluted with PBS and DMEM to 155 μM, and placed at 37 ℃ for different periods. The solutions were filtered to vials with 0.2 μm filters, and subjected to a 1260 HPLC system coupled with a DAD detector. Samples were separated by a C18-EP column (250 × 4.6 mm, 5 μm, Dikma Spursil C18-EP), the mobile phase was 97% A (0.1% formic acid in water, v/v), and 3% B (methanol), the flow rate was 1 mL/min, and the absorbance at 225 nm was detected.

### Cell migration

Transwell assays were conducted using 6-well plates with 50,000 cells seeded in the upper chamber (Costar 3428). The total medium volume with the respective drug was 1 ml per well, while 1 ml of medium with the same drug was placed in the lower chamber. After 12 h of incubation, the cells were fixed with 4% formaldehyde and stained with 0.1% crystal violet. Cells on the upper surface were removed, and 10 fields of view on the lower surface were counted.

For the wound healing assay, cells were seeded in a 6-well plate and allowed to reach 90% confluence. A scratch was made using a 200 μL pipette tip, and the plate was then rinsed three times with PBS to remove detached cells. The remaining cells were incubated in serum-free medium (DMEM, HyClone) at 37 °C. Wound status was recorded at 24 h using an Olympus Corporation microscope. The scratch covered by migrated cells was quantified using ImageJ software (version 1.48; National Institutes of Health, Bethesda, MD, USA).

Live-cell image tracing was conducted by capturing light-field pictures at 12 h intervals. The migration distance of the traced cells was calculated using ImageJ software, with manual correction if necessary.

### Quantification and statistical analysis

Each experiment was repeated at least five times (n ≥ 5), except for sequencing. Statistical analyses were performed using appropriate methods, such as two-tailed t-test, one-way ANOVA, or two-way ANOVA with multiple comparisons, in GraphPad Prism 7.0. The error bars on graphs represent the standard deviation, and the number of independent experiments is denoted by ‘‘n’’. Significance levels are indicated by *, **, and *** representing *P* < 0.05, *P* < 0.01, and *P* < 0.001, respectively, compared to the specified control groups. Complete statistical information can be found in Additional file 4: Table S3.

### Supplementary Information

**Additional file 1: Figure S1.** Derivatives of Asc similarly regulate reprogramming. **Figure S2.** The abilities of Asc metabolites to provide intracellular Asc (Related to Figure 2). **Figure S3.** AscPNa and DHAA regulate the metabolome of MEFs.

**Additional file 2: Table S1.** Gene expression profiles determined in current RNA–seq.

**Additional file 3: Table S2.** The metabolome information collected in the current studies.

**Additional file 4: Table S3.** The detailed statistical information.

**Additional file 5: Table S4.** Reagents and resources used in the current studies.

## Data Availability

The RNA-Seq data results generated during this study are available at Gene Expression Omnibus under accession number GSE108695. All other data generated or analyzed during this study are included in this published article and its Additional file information files.

## Abbreviations

α-KG: α-Ketoglutarate
Asc: l-ascorbic acid
AscNa: l-ascorbic acid sodium salt
AscPNa: l -ascorbic acid trisodium salt
DHAA: l -dehydroascorbic acid
ECAR: Extracellular acidification rate
MET: Epithelial-mesenchymal transition
ERY: l -erythrulose
DKG: 2,3-Diketo-l-gulonic acid
GO: Gene ontology
HIF1α: Hypoxia-inducible factor 1α
iPSCs: Induced pluripotent stem cells
MEFs: Mouse embryonic fibroblasts
MET: Mesenchymal-epithelial transition
OCR: Oxygen consumption rate
OXA: Oxalic acid
OXPHOS: Oxidative phosphorylation
PCA: Principal component analysis
TCA: Tricarboxylic acid
TET1: Ten-eleven translocation methylcytosine dioxygenase 1
THR: l-threonic acid (THR)
