# Supplementary material for: Unraveling the 2,3-diketo-l-gulonic acid-dependent and -independent impacts of l-ascorbic acid on somatic cell reprogramming
Source: Cell Biosci. 2023 Nov 30;13:218. doi: 10.1186/s13578-023-01160-x (PMC10688016; doi:10.1186/s13578-023-01160-x)
Supplement: Supplementary file 1 — Additional file 1: Figure S1. Derivatives of Asc similarly regulate reprogramming. Figure S2. The abilities of Asc metabolites to provide intracellular Asc (Related to Figure 2). Figure S3. AscPNa and DHAA regulate the metabolome of MEFs. [file 13578_2023_1160_MOESM1_ESM.docx]

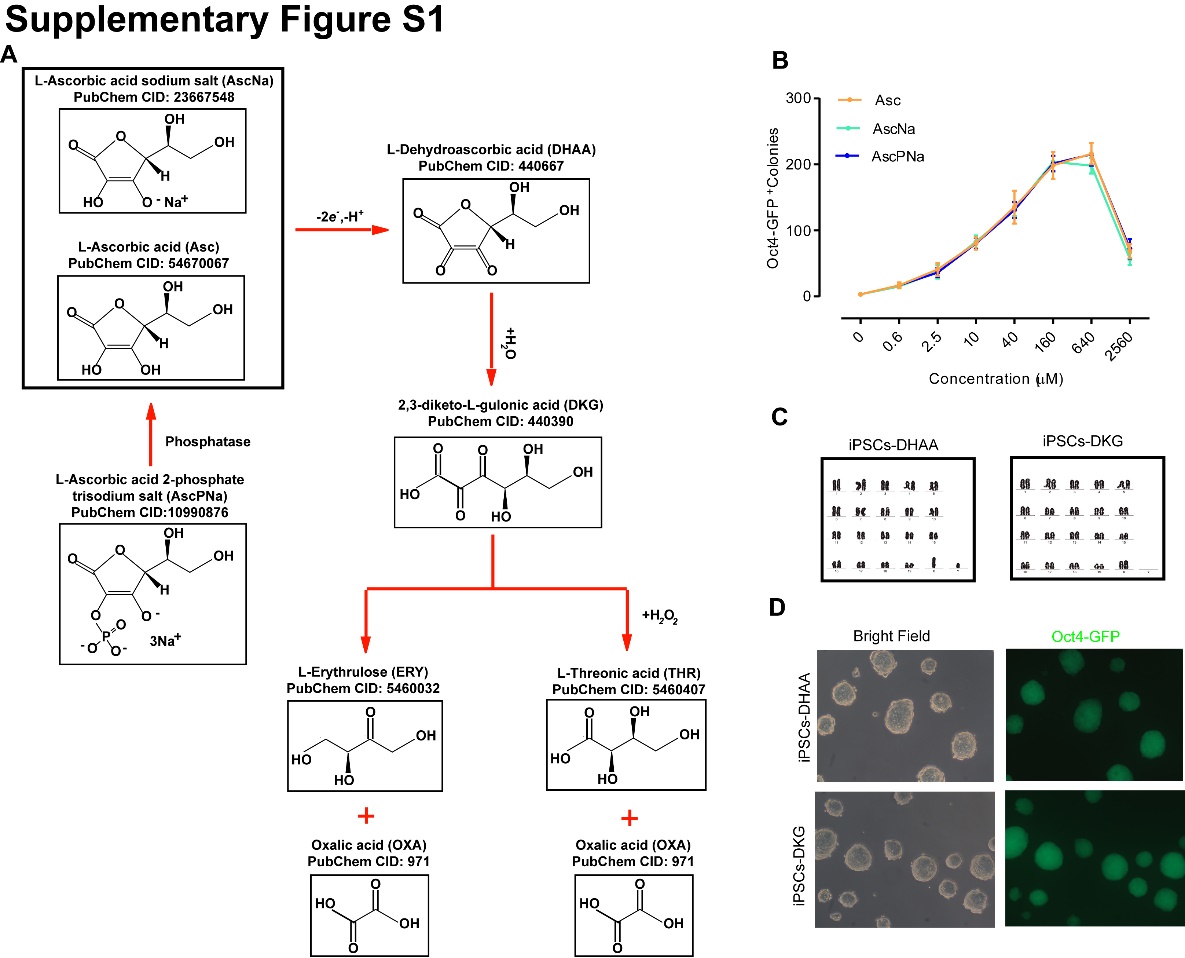


**Additional file 1: Figure S1**

**Derivatives of Asc similarly regulate reprogramming** (Related to Figure 1)

(A) Illustration of the structures of the derivatives (AscPNa and AscNa) and metabolites (DHAA, DKG, ERY, THR, and OXA) of Asc. The metabolic pathway of Asc in mammals is also presented.

(B) Different concentrations of Asc, AscNa, and AscPNa were used during the reprogramming of MEFs. The number of Oct4–GFP^+^ colonies was determined on Day 14.

(C–D) Oct4–GFP^+^ colonies were picked on Day 14 or Day 20 during reprogramming with DHAA and DKG, respectively. The colonies were further cultured and characterized. Karyotypes of the colonies were determined to be normal (C). The morphology of the colonies is represented (D).

All experiments were repeated at least five times (n≥5). Additional statistical information is listed in Table S3.


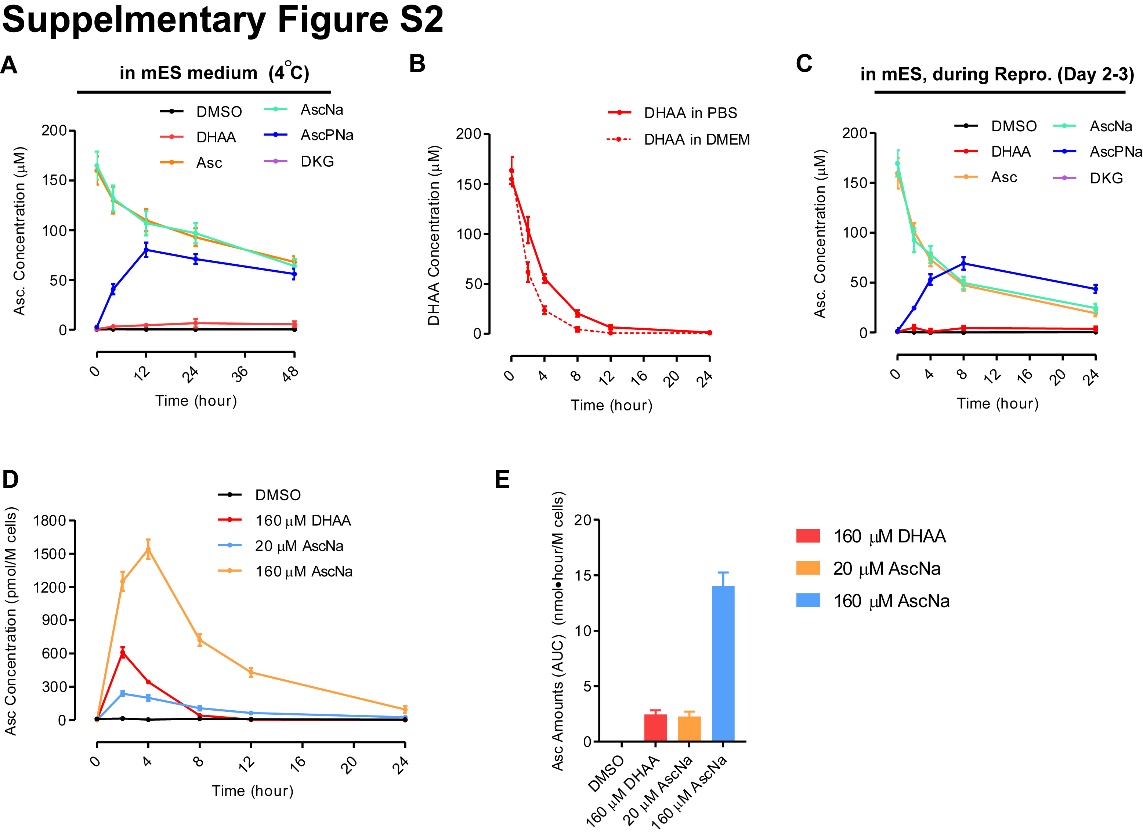


**Additional file 1: Figure S2**

**The abilities of Asc metabolites to provide intracellular Asc (Related to Figure 2)**

(A) 160 μM AscPNa, AscNa, Asc, DHAA, and DKG were stored in mES medium for 48 hours. The concentration of Asc in the mES medium was determined.

(B) 160 μM DHAA was prepared in PBS or in DMEM and stored at 37°C. The concentration of DHAA was determined by HPLC.

(C) Reprogramming with 160 μM AscPNa, AscNa, Asc, DHAA, or DKG. The extracellular Asc (in mES medium) concentration was measured in the 24 hours after switching to fresh mES medium at the beginning of day 3 (A).

(D–E) Indicated concentrations of AscNa and DHAA were used to treat MEFs, and the concentration of intracellular Asc was determined at different time points (D). The accumulated amount of intracellular Asc was also calculated (E).

All experiments were repeated at least five times (n≥5). Additional statistical information is listed in Table S3.

**
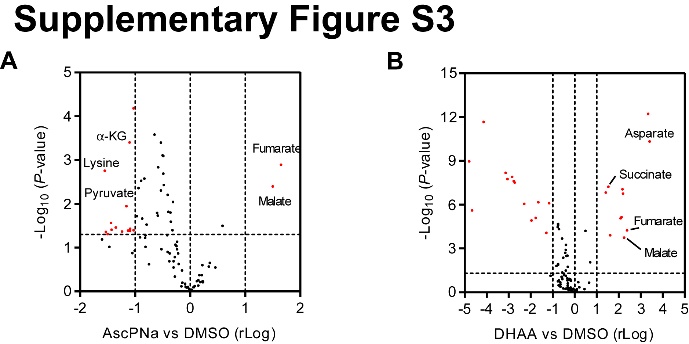
**

**Additional file 1: Figure S3**

**AscPNa and DHAA regulate the metabolome of MEFs**

(A) The compounds regulated by AscPNa were identified with a volcano plot.

(B) The compounds regulated by DHAA were identified with a volcano plot.

Metabolome analysis was performed at least eight times (n≥8). Additional statistical information is listed in Table S3.

**Table S1**

**Gene expression profiles determined in current RNA–seq**

DMSO, 160 μM AscPNa, and 160 μM DKG were used during the reprogramming. RNA–seq was performed on Day 3 and Day 8 during the reprogramming. The original sequencing results were deposited under accession number GSE108695. The normalized results were provided in the current table.

**Table S2**

**The metabolome information collected in the current studies**

As title.

**Table S3**

**The detailed statistical information**

As title.

**Table S4**

**Reagents and resources used in the current studies**

As title.
